# Restless leg syndrome in chronic hemodialysis patients in Mashhad hemodialysis centers

**DOI:** 10.15171/jrip.2017.27

**Published:** 2016-12-25

**Authors:** Niloufar Zadeh Saraji, Maryam Hami, Reza Boostani, Mohammad Javad Mojahedi

**Affiliations:** ^1^Kidney Transplantation Complications Research Center, Mashhad University of Medical Sciences, Mashhad, Iran; ^2^Department of Neurology, Ghaem Hospital, Mashhad University of Medical Sciences, Mashhad, Iran

**Keywords:** Restless leg syndrome, Hemodialysis, Chronic kidney disease, Hemodialysis, End-stage renal disease

## Abstract

**Introduction:** Restless leg syndrome (RLS) is a sensory motor disorder. Patients with this syndrome have serious and uncontrollable desire to move their legs, which is mostly due to an uncomfortable feeling intensified when they are motionless. It may be a genetic disorder or secondary to iron deficiency, neurodegenerations, pregnancy, some drugs and severe kidney diseases.

**Objectives:** This study was designed to find out the prevalence and its risk factors of RLS in hemodialysis patients.

**Patients and Methods:** This multicenter cross-sectional study was done on 260 hemodialysis patients. The prevalence of RLS was measured using International Restless Legs Syndrome Study Group (IRLSSG)’s RLS Questionnaire (RLSQ). Potential risk factors for RLS including underlying cause of chronic renal failure, duration on dialysis, biochemical tests, dialysis adequacy, and erythropoietin and also venofer dosage in recent month and demographic data were also evaluated.

**Results:** The prevalence of RLS was 55% including 59.4% males and 40.6% females. Their mean age of RLS patients and their dialysis duration were significantly higher than other group (*P*<0.05). Their body mass index (BMI) and serum calcium were significantly higher (*P*<0.05). However erythropoietin dosage and serum hemoglobin level were lower in RLS patients (*P*<0.05). Significant predictors of RLS were history of diabetes mellitus (DM), hypertension (HTN), smoking (*P*<0.05). There was not significant relation between RLS and dialysis adequacy, serum intact parathyroid hormone (iPTH), urea, ferritin and venofer dosage (*P*>0.05).

**Conclusion:** According to the results, RLS is a common disorder in hemodialysis patients which can affect strongly on their life. So particular attention and sooner diagnosis of RLS in high risk patients for better management is necessary.

**Implication for health policy/practice/research/medical education:**

In a multicenter cross-sectional study on 260 stable hemodialysis patients to detect the prevalence of RLS and its risk factors, we found its prevalence was 55% of patients. It is appeared RLS is a common disorder in hemodialysis patients and patients with past history of DM, HTN and smoking are more prone to it. So particular attention and sooner diagnosis of RLS in high risk patients for better management is necessary.

## Introduction

Restless leg syndrome (RLS) is a sensorimotor disturbance with features of both neurologic and sleep disorders. Patients who afflicted describe an intensely uncomfortable urge to move legs predominantly in the evening or at night, which disturbs their sleep. The pattern of movement is involuntary dorsiflexion of foot and lower leg, that lasting 2 to 5 seconds (1-5).

RLS is described in two form of primary and secondary. Primary RLS occurs in patient with positive family history who is usually older than 45 years (2). Secondary RLS can be happened in patients with uremia, pregnancy, iron deficiency state, rheumatoid arthritis, diabetes mellitus (DM) type 2 and some neuropathies (2). In primary RLS, dysfunction of the dopaminergic system and reduced iron stores in specific regions in the brain are implicated as pathophysiologic mechanism, however in secondary RLS in uremic patients in spite of dysfunction of dopaminergic system, calcium and phosphate imbalance can help to its initiation and also its severity (6).

RLS is important as it can affect on sleep quality and cardiovascular function, as it can accelerate nocturnal hypertension (HTN), stroke, depression and anxiety in patients (2). It is appeared hemodialysis patients who suffered from RLS, have higher mortality rate than other end-stage renal disease (ESRD) patients (7).

## Objectives

Based on previous studies, prevalence of RLS in general population is between 2.5% and 15%, which increases with age and probably higher in female gender. Its prevalence is even more in dialysis patients especially when using International Restless Legs Syndrome Study Group (IRLSSG), that are reported about 20% to 62% (8). Hence, we tried to find prevalence and related factors in ESRD in hemodialysis centers in Mashhad.

## Materials and Methods

We conducted an observational cross-sectional study on 260 consecutive hemodialysis patients from October to December 2014 at Ghaem, Imam Reza, Montaseriye, Mousabenejafar hospitals, and kidney diseases organization hemodialysis centers in Mashhad, Iran. We enrolled all stable patients were older than 16 year-old with at least 6 months history of chronic hemodialysis in above centers. Patients who were not cooperative, like patients with confusion or dementia were excluded. Also patients taking regular dosage (more than 10 times per month) of anti-histaminic drugs, dopamine agonists, tricyclic antidepressant, benzodiazepines, gabapentin, anti-convulsion and alcohol consumption were excluded. The data collection was carried out by personal interview, using standardized diagnostic questionnaire by IRLSSG. Patients were evaluated by face-to-face interviews. The questionnaire was also consisted of demographic and laboratory data such as age, gender, weight, height, marital status, employment, medication, smoking and coffee or tea intake, the underlying cause of chronic renal failure, duration on dialysis, intravenous iron dosage and erythropoietin dosage in recent month, serum hemoglobin, serum ferritin, serum urea, calcium and phosphate and also dialysis adequacy (Kt/V).

### Ethical issues

The research followed the tenets of the Declaration of Helsinki. Informed consent was obtained and the research was approved by the Ethics Committee of Mashhad Univer­sity of Medical Sciences.

### Statistical analysis

Statistical analysis was performed by the SPSS software version 21. Categorical variables were expressed as proportions and compared with the chi-square test. Variables such as age and body mass index (BMI) were expressed as means and standard deviations, and were compared with *t* tests. *P* value less than 0.05were considered statistically significant.

## Results

The total number of patients recruited for the study was 260. The mean age was 48.99± 15.72 years. There were 157 males (60.4%) and 103 females (39.6%). The mean duration on dialysis was 47.88 ± 40.44 months. The commonest etiology of chronic kidney disease was HTN in 78 (30%) patients and DM in 73 (28.1%) patients. The majority of patients (81.9%) were married and most of them (85.8%) were unemployed. The mean BMI was 22.91± 4.22 kg/m^2^. Other demographic and laboratory data are shown in Table 1.

**Table 1:** Demographic and laboratory data in patients

**Characteristics**	**RLS**	**No RLS**	***P***
M/F	85/58	72/45	0.731
Age (y)	51.72±14.49	45.65±16.57	0.002
Weight (kg)	62.94±13.02	59.91±12.32	0.057
Height (cm)	162.42±16.47	163.90±9.25	0.386
BMI(kg/m^2^)	23.48±4.34	22.22±3.99	0.017
Dialysis duration (y)	4.72±5.03	3.10±4.53	0.008
PTH (ng/L)	485.28±451.85	476.22±425.96	0.869
Ferrittin (ng/mL)	1107±07	490.80±428.83	0.093
Hemoglobin (g/ dL)	11.04±1.98	11.70±2.74	0.031
Urea (mg/dL)	126.78±34.95	128.72±37.03	0.665
Calcium(mg/dL)	8.43±0.86	8.20±0.82	0.034
Phosphosrus (mg/dL)	5.79±1.37	5.54±1.68	0.187
Kt/V	1.36±0.33	1.43±0.36	0.113
EPO dosage (unit/wk)	7804.19±6610.12	10111.11±6543.98	0.005
Venofer dosage (mg/mon)	531.5±0.50	555.6±0.49	0.699

The study results indicated that out of 260 hemodialysis patients 143 (55%) presented RLS. There were significant relation between causes of kidney disease and RLS, while, patients with history of DM and HTN had significantly higher frequency of RLS (*P = *0.001). However patients with past history of urologic problems and rheumatologic disease had not this relation (*P* > 0.05). Detail of underlying disease has been compared in patients with and without RLS in Table 2. Totally 72 (27.8%) of patients were retired. 49 (68.1%) of them had RLS that was significantly higher percentage than other groups (*P = *0.035) that consist of employed and unemployed patients. In employed group, we did not find significant relation between job and RLS. Married patients had significantly higher symptoms of RLS, as 126 (59.4%) of 212 married patients had RLS. However in unmarried group 16 (34%) of 47 patients had RLS (*P = *0.004). While only 12 (4.6%) patients were smoker, most of them had RLS (83.3%) (*P = *0.043). Patients evaluated for tea consumption, there was not significant relation between frequency of RLS and daily tea consumption (*P = *0.165). There were no significant differences in prevalence of RLS in patients who took eligible dosage (less than 10 times in month) of antihistaminic drugs, anticonvulsant drugs and tricyclic antidepressant drugs (*P > *0.05). However, in RLS patients gabapentin consumption was more than other patients (*P = *0.02; Table 3).

**Table 2:** Underlying disease and RLS

	**RLS**	**No RLS**
HTN	46 (59.1%)	31 (40.3%)
DM	26 (81.2%)	6 (18.8%)
Diabetes + HTN	27 (67.5%)	13 (32.5%)
Urologic diseases	17 (36.2%)	30 (63.8%)
Trauma	2 (33.3%)	4 (66.7%)
Other diseases	23 (41.1%)	33 (58.9%)

**Table 3:** Drug history and RLS

**Drug**	**RLS**	**No RLS**	***P*** ** value**
Anti-histaminic drug	32(22.4%)	22(18.8%)	0.48
SSRI	16(11.2%)	11(9.4%)	0.638
Benzodiazepines	22 (15.4%)	11(9.4%)	0.149
Anticonvulsant	9 (6.3%)	8(6.8%)	0.86
TCA	13(9.1%)	5(4.3%)	0.128
Gabapentin	30(21%)	10(8.5%)	0.006

## Discussion

This is the first study of prevalence of RLS in hemodialysis patients in Mashhad, north-east of Iran. Previously Zamani et al reported prevalence of RLS in general population in Tehran (Iran) in patients referring to neurology and orthopedic clinics, which was 10.5% (9). Based on a systematic review in 2010, Innes et al reviewed 34 papers about prevalence of RLS in western countries. They reported prevalence rates ranged from 4% to 29% of adults across studies (10).

It is assumed RLS is more common in hemodialysis patients, as uremia is one of the causes of secondary RLS. Chavoshi et al reported the prevalence of RLS was about 31.7% in hemodialysis patients in Tehran, Iran (2). Al-Jahdali et al evaluated 227 ESRD patients in Jeddah, Saudi Arabia. The prevalence of RLS was 50.22% (11). Similar studies in other countries reported prevalence of RLS to be 6% to 80% in patients with chronic renal failure (12-15), for example 21.5% in Brazil (16), 14% in Canada (17), 18.4-21.5% in Italy (18), 6.6% in India (19), 20.3% in Syria (20), 62% in China (21), and 12%-23% in Japan (22,23). These highly variable results may be due to difference in races, diagnostic criteria of RLS, methods of studies. Our results were in line with Saudi Arabia and China studies.

In this study we did not find any association between gender and RLS. However some previous studies (2,10,11) found this relationship. It can be explained by lower proportion of patients.

The mean age of patients with RLS was significantly higher than other group that was similar to previous studies, that RLS were happened more in older dialysis patients (2,10,11). It can be confirmed by higher RLS in retired patients.

Our results revealed that BMI of patients with RLS was significantly higher than patients without RLS. This is compatible with the results of Giannaki et al (6) and Bayard et al (24) that reported RLS was more prevalent in overweight patient. Even it is suggested that weight loss can help to symptom relieve of RLS. Although some other studies did not approve this relationship (2,11).

Based on our results, longer dialysis duration was associated with higher prevalence of RLS. These results are similar to results of Giannaki et al (6) and Gigli et al (4). However other studies (2,11) could not find this association. It can be explained that neuropathy in ESRD patients appears as a late symptom and can be deteriorated by hemodialysis as therapeutic method.

We did not find any difference between two groups regarding serum PTH, phosphorus level and RLS syndrome. However difference in serum calcium was significant. Moreover, calcium/phosphate imbalance is also reported to be involved in the pathophysiology of uremic RLS (23). It can be approved by more studies.

Results showed there was no correlation between serum urea and dialysis adequacy (Kt/V) and RLS. Previous study showed this association is controversial (6). Hence, it is necessary to conduct larger studies on this aspect of dialysis patients.

Some studies revealed that iron deficiency can lead to RLS, even in the absence of anemia (25). In contrast with Sloand et al, we did not find any association between serum ferritin levels and RLS. However we found significant correlation of serum hemoglobin level and RLS. It can be explained by that serum ferritin level may be affected by other factors such as inflammation, and is not good predictor of iron store (26). Erythropoietin dosage was significantly lower in RLS group. Thus we concluded that, correction of anemia by higher dosage of erythropoietin can decrease or resolve RLS symptoms in dialysis patients.

Accordingly, patients with past history of DM had significantly higher prevalence of RLS. Previous studies suggested DM is a dependent risk factor of RLS (11). We found also a significant relation between HTN and RLS that similar results observed before (27).

Chen et al found that smoking was associated with RLS, whereas consumption of coffee and tea had a negative effect on RLS (28). Gigli et al did not find any association between smoking, coffee intake and RLS (4). Al-Jahdali et al found significant correlation between RLS and daily regular use of coffee, but not with cigarette smoking (11).

We found significant relation between smoking and RLS, but not tea consumption.

Our RLS patients took anticonvulsant gabapentin significantly higher than non-RLS group. It can be explained by effects of Gabapentin on RLS symptoms which relives after it’s taking (6). Other drugs that effect on RLS severity were similar in both groups.

## Conclusion

Using the IRLSSG questionnaire to identify dialysis patients with RLS, the prevalence was significantly high. RLS may result poor quality of life. Therefore, physicians taking care of dialysis patients should be aware of RLS to diagnose it as soon as possible. Future research should focus on unfolding the mechanisms underlying the high prevalence of RLS in the uremic patients and better treatment of it.

## Limitations of the study

Some limitations of our study were selection bias from collecting data from only selected dialysis centers; also we did not obtain data on nerve conduction parameters.

## Acknowledgments

This study was the result of the MD thesis of Niloufar Zadeh Saraji.

## Authors’ contribution

NZS; acquisition of data and analysis and interpretation of them. MH; conception and design, drafting the article and revising it. RB; conception and design. MJM; final approval of the version.

## Conflicts of interest

The authors declare no conflict of interests.

## Ethical considerations

Ethical issues (including plagiarism, data fabrication, double publication) have been completely observed by the authors.

## Funding/Support

The authors would like to thank the Office of Vice Chancellor for Research of Mashhad University of Medical Sciences for financial support of this study (Grant # 89148).
